# A systematic review and meta-analysis of HIV associated neurocognitive disorders (HAND) among people with HIV in Ethiopia

**DOI:** 10.1186/s12981-021-00424-1

**Published:** 2021-12-19

**Authors:** Yosef Zenebe, Baye Akele, Mulugeta W/Selassie, Mogesie Necho

**Affiliations:** 1 Department of Psychiatry, College of Medicine and Health Sciences, Wollo University, Dessie, Ethiopia; 2 Department of Pharmacy, College of Medicine and Health Sciences, Wollo University, Dessie, Ethiopia; 3 Department of Pediatrics and Child Health Nursing, College of Medicine and Health Sciences, Wollo University, Dessie, Ethiopia

**Keywords:** Ethiopia, Systematic review, HAND, HIV/AIDS

## Abstract

**Background:**

Ethiopia, being in the Sub Saharan region of Africa, is one of the countries with a substantial burden of HIV infection. Because of the high burden of HIV and poor health care settings, HAND is prevalent as demonstrated in various cross-sectional studies. However, no review has been conducted to report the consolidated magnitude of HAND among people with HIV in Ethiopia. Therefore, this systematic review and meta-analysis aimed to estimate the prevalence of HAND in Ethiopia.

**Methods:**

Following the PRISMA guidelines, we systematically reviewed and meta-analyzed studies that investigated the prevalence of HAND in Ethiopia from PubMed, Google Scholar, Science Direct, HINARI, EMBASE, and Cochrane library databases. We also looked at the reference lists of the included studies to include other relevant studies. Subgroup analysis was performed based on publication year, study location, and sample size. Heterogeneity across studies was evaluated using the I2 test. Potential publication bias was assessed using Egger’s test and visual inspection of symmetry in the funnel plots.

**Results:**

In the present meta-analysis, 627 articles were initially identified and evaluated. Of these, 8 studies that met the inclusion criteria were included in the final analysis. The pooled prevalence of HAND in people with HIV in Ethiopia was 39.15% (95% CI 29.36, 48.94). The highest prevalence observed in the Southern Nations, Nationalities, and Peoples’ Region (SNNPR) with 53.20% (95% CI 25.96, 80.44) followed by others 34.87% (Tigray, Addis Ababa, and Oromia) (95% CI 33.49, 36.24) and Amhara 34.07% (95% CI 25.39, 42.74).The funnel plot was asymmetrical. However, Egger’s regression tests provided no evidence of publication bias in the prevalence of HAND.

**Conclusion:**

In this meta-analysis, the pooled prevalence of HAND, in Ethiopia, was high. Older age, substance use, advanced stages of the disease, and lack of education were the main determinants of HAND in Ethiopia. Health education, early screening of people with HIV, and training of health professionals working in hospitals on HAND are highly recommended.

## Background

Human immunodeficiency virus (HIV) is neuro-virulent and often causes brain impairment, especially at advanced stages of HIV infection. Subcortical brain structures are the most frequently affected areas of the brain by HIV; thus the resulting changes in these structures ultimately cause a spectrum of disorders that are collectively referred to as HAND [1, 2]. HAND covers three disorders ranging in severity from asymptomatic neurocognitive impairment (ANI) to mild neurocognitive disorder (MND) to HIV-associated dementia (HAD) [3–7].These disorders have a negative impact not only on routine daily activities such as cleaning, cooking, money calculation, driving, etc. but also on adherence to treatment and social and professional integration [8, 9].

Africa, especially its sub Saharan region, is one of the continents that are sternly affected by HIV. Ethiopia, being in the sub-Saharan region, is one of the countries with a substantial burden of HIV infection [10]. Because of the high burden of HIV and poor health care settings, HAND is prevalent as demonstrated in various cross-sectional studies. A study conducted in Addis Ababa by Araya et al. [11] revealed that 35.6% of the study participants had developed HIV-associated neurocognitive disorder (HAND). Another study conducted at Ayder Hospital in the Tigray region of Ethiopia demonstrated that 33.3% of HIV-positive Adults on ART experienced HAND. A few more cross-sectional facility-based studies have failed to map the real burden of HAND [12].

There are many predictors of HAND in patients with HIV infection. These include; CD4 count, the onset of opportunistic infection, low hemoglobin concentration, Body mass index, advanced age, female sex, level of education, income level, social support, medical comorbidities, intravenous drug use, HIV medication adherence, self-reported alcohol use, khat chewing, lifetime use of tobacco, marital status and unemployment status [11, 13–21].

Although few facility-based cross-sectional studies have been conducted previously in different parts of Ethiopia on HAND, the pooled prevalence of the problem in Ethiopia is unknown. Additionally, the risk factors reported by different researchers have been inconsistent. This might be due to social, cultural, and lifestyle differences among the study subjects across the country. This systematic review and meta-analysis aimed to determine the pooled prevalence of HAND in Ethiopia and the determinant factors influencing it. This study may also draw a new hypothesis based on this heterogeneity. Hence, this research is of paramount importance in providing clear crystal evidence to policymakers, researchers, and clinicians to ease their work on HAND.

## Methods

### Search strategy

The present systematic review and meta-analysis were conducted based on a review of different types of literature. The international databases including PubMed, Google Scholar, Science Direct, HINARI, EMBASE, and Cochrane library were exhaustively searched. In addition, reference lists of previously identified articles were also searched to retrieve more relevant studies. Preferred Reporting Items for Systematic Reviews and Meta-Analyses (PRISMA) [22] was used as a guideline for rigor. The search was carried out using the following keywords by the Boolean operator: “Prevalence” OR “Epidemiology” AND “neuro-cognitive impairment” OR “HIV Associated Neurocognitive Disorders” OR “HAND” OR “Neurocognitive Disorders” OR “Cognitive disorders” OR “Cognitive impairment” AND “HIV/AIDS” AND “Ethiopia.” The included studies were published from January 2014 to April 16, 2021.

### Eligibility criteria

All original studies published until April 16, 2021, were included in this review. An article was included if it met the following criteria: (1) the study was conducted in Adults, (2) the study design was observational (cross-sectional and case–control study design), (3) the outcome of interest was HAND, (4) human studies and (5) conducted in Ethiopia. We excluded editorials and reviews.

### Data extraction

Extractions of the required data from the studies and the full texts of the articles were assessed by two authors (YZ and MN). Any discrepancy was resolved by discussion, and the following information was independently extracted from each study by two authors (YZ and MN) using a standardized data extraction format: primary author, publication year, region of the study (study site in the country), sample size, screening tool used, response rate and reported prevalence of HAND.

### Quality assessment

The Joanna Briggs institute quality assessment tool was used to assess the quality of the studies included in this meta-analysis [23]. The scoring of each publication was performed using the frequency scales that were answered as yes, no, unclear, and not applicable. The total quality score for each study was calculated based on the total number of positive scores.

### Statistical analysis

In the current meta-analysis, all statistical analyses were conducted using comprehensive meta-analysis software version 3 [24]. The prevalence rates from individual studies were pooled using a random-effects meta-analysis [25]. I2 statistics have been used to assess heterogeneity between studies [25]. The values of I2 statistics such as75%, 50%, and 25%, represented high, medium and low heterogeneity respectively [26]. The publication year, study location and sample size of the studies were used to evaluate the possible sources of heterogeneity across the studies. Publication bias was assessed using funnel plots and Egger’s regression tests. For all analyses, the P-value for statistical significance was set at 0.05.

### Definition of terms

Advanced stages of the diseases: People living with HIV who were in WHO clinical stage T3 and T4.

HAND: It included three disorders such as asymptomatic neurocognitive impairment (ANI), mild neurocognitive disorder (MND) and HIV-associated dementia (HAD) [3–5, 27, 28].

Older age: People living with HIV whose age was 40 years or more.

## Results

### Search result

**Fig. 1:**
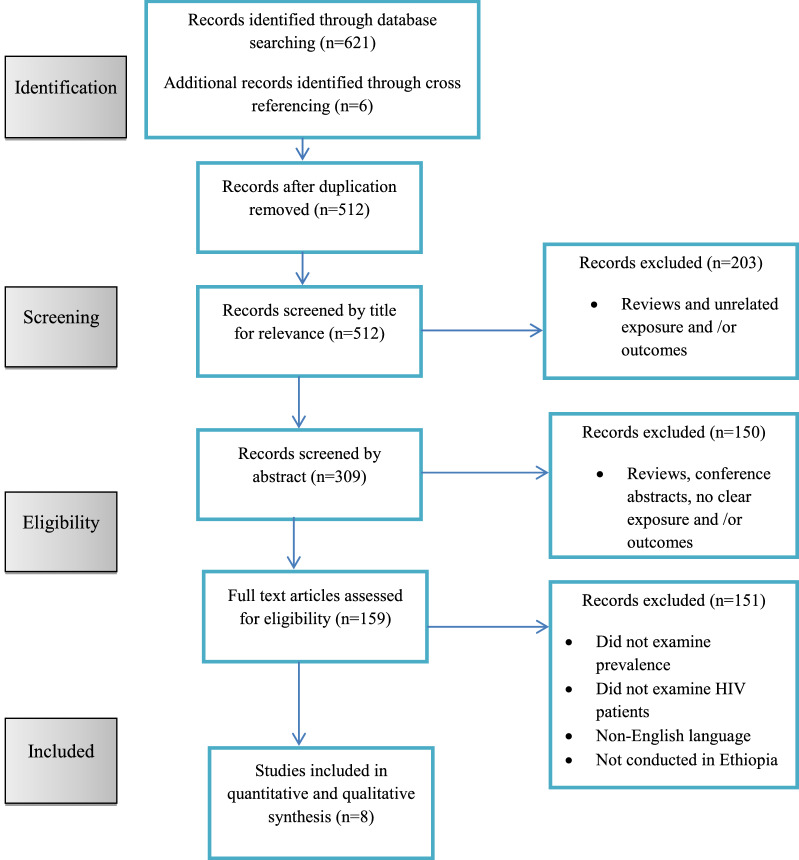
Flow diagram of included studies

The electronic database search retrieved 627 records, of which 115 were duplicates. The titles and abstracts of 512 articles were assessed, and 353 articles were removed. Therefore, a full-text of 159 publications was retained for further evaluation, 8 of which were qualified for the present systematic review and meta-analysis (Fig. 1).

### Characteristics of studies included

In this review, a total of 8 studies were included in the final meta-analysis conducted in five regions in Ethiopia representing 3529 participants. Of the 8 studies, three were published from 2014 to 2017 years [12, 15, 28] and five were published from 2019 to 2021 years [11, 21, 29–31]. Three studies were from Tigray, Addis Ababa, and Oromia [11, 12, 31], three were from Amhara [15, 28, 30] and two were from SNNPR [21, 29]. Three studies used a sample size of less than 400 [12, 29, 31], and five of the studies used a sample size of more than 400 [11, 15, 21, 28, 30] (Table 1).

**Table 1:** The characteristics of studies included in the systematic review and meta-analysis of HAND in Ethiopia

Author, publication year	Location of the study	Study setting	Study design	Sample size	Tool used	Prevalence	Number of cases
Belete et al. 2017	Mekelle, Ethiopia	Health institution	CS	254	IHDS ≤ 9.5	33.3%	85
Tsegaw et al. 2017	South Wollo, Ethiopia	Health institution	CS	593	IHDS ≤ 9.5	36.4%	216
Animut et al. 2019	Gamo Gofa, Ethiopia	Health institution	CS	684	IHDS < 9.5	67.1%	459
Araya et al. 2020	Addis Ababa, Ethiopia	Health institution	CS	581	IHDS ≤ 9.5	35.6%	207
Yitbarek et al. 2019	Jimma, Ethiopia	Health institution	CS	328	IHDS ≤ 10	35.7%	117
Mossie et al. 2014	Debre Markos, Ethiopia	Health institution	CS	423	IHDS ≤ 10	24.8%	105
Wubetu et al. 2021	Debre Berhan, Ethiopia	Health institution	CS	422	MMSE < 25	41%	173
Salahuddin et al. 2020	Mizan-Aman, Ethiopia	Health institution	CS	244	IHDS ≤ 10	39.3%	96

*CS* cross-sectional, *HAND* HIV Associated Neurocognitive Disorders, *IHDS* International HIV Dementia Scale, *MMSE* Mini Mental State Examination

### Quality of included studies

Based on the Joanna Briggs institute quality evaluation checklist, the articles involved in the final analysis had a mean quality score of 8.75 ranging from seven to nine. Seven studies (87.5%) were high-quality studies (score ≥ 8.75) and the remaining one was moderate quality article (scored between 6 and 8.75). None of the articles were found to be of poor quality (Table 2).

**Table 2:** Qualities of studies included in the systematic review and meta-analysis

Study name	Response									
	Q1	Q2	Q3	Q4	Q5	Q6	Q7	Q8	Q9	Total
Belete et al. 2017	Y	Y	Y	Y	Y	Y	Y	Y	Y	9
Tsegaw et al. 2017	Y	Y	Y	Y	Y	Y	Y	Y	Y	9
Animut et al. 2019	Y	Y	Y	Y	Y	Y	Y	Y	Y	9
Araya et al. 2020	Y	Y	Y	Y	Y	Y	Y	Y	Y	9
Yitbarek et al. 2019	Y	Y	Y	Y	Y	Y	Y	Y	Y	9
Mossie et al. 2014	Y	Y	Y	Y	Y	Y	Y	Y	Y	9
Wubetu et al. 2021	Y	Y	Y	Y	Y	Y	Y	Y	Y	9
Salahuddin et al. 2020	Y	N	U	Y	Y	Y	Y	Y	Y	7

Q1–Q9 represents questions used to assess the quality of included studies, which are listed below

Q1. Was the sample frame appropriate to address the target populations?

Q2. Were the study participants sampled in appropriate way?

Q3. Was the sample size adequate?

Q4. Were the study subjects and setting described in details?

Q5. Was the data analysis conducted with sufficient coverage of the identified sample? Q6. Was a valid method used in the identification of conditions?

Q7. Was the condition measured in a standard, reliable way for all participants?

Q8. Was there an appropriate statistical analysis?

Q9. Was the response rate adequate, and if not, was the low response rate managed appropriately?

*N* no, *NA* not applicable, *U* unclear, *Y* yes

### The prevalence of HAND among People with HIV

**Fig. 2:**
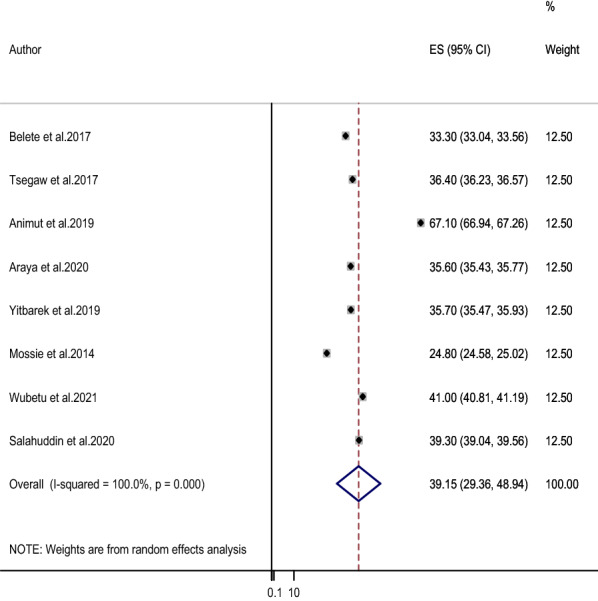
The forest plot of the prevalence of HAND among People with HIV in Ethiopia: a meta-analysis

There was significant heterogeneity between the studies (I2 = 100%, P < 0.001), therefore, we used random-effects models to estimate the prevalence of HAND in people with HIV. The overall pooled estimated (random effects models) of the prevalence of HAND in people with HIV was 39.15% (95% CI 29.36–48.94) (Fig. 2). The pooled estimate of the prevalence was higher in the SNNPR region than in the Amhara region (53.20%; 95% CI 25.96, 80.44 vs. 34.07%; 95% CI 25.39, 42.74) (Table 3).

**Table 3:** A subgroup analysis among people living with HIV

Subgroup	Number of studies	Estimates		Heterogeneity	
		Prevalence (%)	95% CI	I 2	P-value
Year of publication					
2014–2017 Years	3	31.50	24.32, 38.68	100%	< 0.001
2019–2021 Years	5	43.74	30.32, 57.16	100%	< 0.001
Location of the study					
Amhara	3	34.07	25.39, 42.74	100%	< 0.001
SNNPR	2	53.20	25.96, 80.44	100%	< 0.001
Others (Tigray, Addis Ababa and Oromia)	3	34.87	33.49, 36.24	99.2%	< 0.001
Sample size					
Below 400	3	36.10	32.83, 39.37	99.8%	< 0.001
Above 400	5	40.98	26.64, 55.32	100%	< 0.001

### Subgroup analysis among people living with HIV

In addition, subgroup analysis was performed based on the year of publication, location of the study, and sample size. Regarding the publication year, the prevalence of HAND was slightly higher in studies published from 2019 to 2021 years 43.74% (30.32, 57.16) followed by studies from 2014 to 2017 years 31.50% (24.32, 38.68). Accordingly, the highest prevalence was observed in SNNPR with a prevalence of 53.20% (25.96, 80.44) followed by others (Tigray, Addis Ababa, and Oromia) (34.87%, 33.49, 36.24) and the Amhara region (34.07%, 25.39, 42.74).The prevalence of HAND was 40.98% (95% CI 26.64, 55.32) for studies that used a sample size of more than 400 and it was 36.10% (95% CI 32.83, 39.37) for studies that used a sample size of less than 400 (Table 3).

### Sensitivity analysis of HAND among people living with HIV

**Fig. 3:**
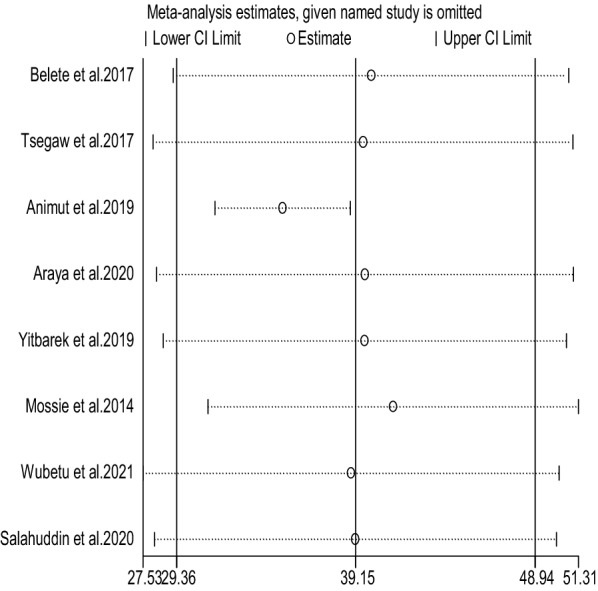
Sensitivity analysis for the prevalence of HAND among people with HIV in Ethiopia

Sensitivity analysis was performed to identify whether one or more of the eight studies had out-weighted the average prevalence of HAND among people living with HIV. However, the result showed that there was no single influential study since the 95% CI interval obtained when each of the eight studies was excluded at a time is within the 95% CI interval of the overall result (Fig. 3).

### Funnel plot of the risk of publication bias for HAND among people with HIV

**Fig. 4:**
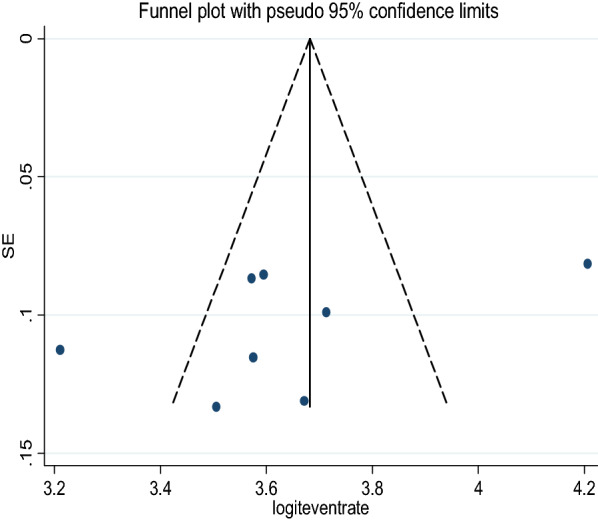
Funnel plot of standard error by logit event rate (publication bias)

Qualitatively, the funnel plot was asymmetric, supporting the presence of publication bias by visual inspection. However; quantitatively, Egger regression tests provided no evidence of substantial publication bias for the prevalence of HAND among people with HIV in Ethiopia (P = 0.201) (Fig. 4).

### A systematic review and meta-analysis of associated factors for HAND in Ethiopia

Older age, having no education (being illiterate), having a primary education, being unemployed, having a low income, having a body mass index of 16 kg/m^2^, poor social support, substance use, having a comorbid opportunistic infection, comorbid depression, anxiety, and advanced disease stage were significant and positive predictors of HAND (Table 4). The factors most frequently associated with HAND in People with HIV were older age [15, 28–31], being illiterate [11, 15, 28], substance use [11, 15, 29, 31], and advanced stage of the disease [12, 21, 31].

**Table 4:** A systematic review and meta-analysis of associated factors for HAND in Ethiopia

Associated factors	Odds ratio(AOR)	95% confidence interval	Strength of association	Author, year of publication
Older age	3.31	1.26, 8.70	Strong and positive	Tsegaw et al. 2017
Age group of 41–64 years	3.1	1.3, 7.4	Strong and positive	Yitbarek et al. 2019
Older age of 50 years or above	4.25	1.05, 17.18	Strong and positive	Mossie et al. 2014
Older individuals	1.06	1.03, 1.08	Weak and positive	Wubetu et al. 2021
Being older than 40 years	2.81	1.11, 7.15	Moderate and positive	Salahuddin et al. 2020
No formal education	4.29	2.62, 7.02	Strong and positive	Tsegaw et al. 2017
Being illiterate	5.16	2.20, 12.07	Strong and positive	Araya et al. 2020
Having no education	3.11	1.37, 7.04	Strong and positive	Mossie et al. 2014
Having a primary-level education	3.27	1.46, 7.29	Strong and positive	Araya et al. 2020
Being married	0.377	0.21, 0.67	Weak and negative	Animut et al. 2019
Unemployment status	3.18	1.75, 5.78	Strong and positive	Animut et al. 2019
Low monthly income	4.22	2.02, 8.81	Strong and positive	Wubetu et al. 2021
Body mass index 16 kg/m^2^	4.39	1.60, 12.02	Strong and positive	Animut et al. 2019
Having poor social support	3.65	1.86, 7.17	Strong and positive	Wubetu et al. 2021
Poor medication adherence	1.49	1.01, 2.18	Weak and positive	Tsegaw et al. 2017
Being non-compliant with prescribed medications	2.99	1.01, 8.87	Moderate and positive	Salahuddin et al. 2020
Lifetime use of tobacco	2.40	1.44, 4.01	Moderate and positive	Araya et al. 2020
Khat chewing	4.4	2.3, 8.3	Strong and positive	Yitbarek et al. 2019
Substance use	4.64	2.3, 9.36	Strong and positive	Mossie et al. 2014
Having a history of recreational drug use	13.67	6.42, 29.13	Strong and positive	Salahuddin et al. 2020
CD4 count of 500 cells/dl or less	2.37	1.52, 3.68	Moderate and positive	Tsegaw et al. 2017
Having a CD4 count (cells/μl) ≤ 500	1.61	1.11, 2.39	Weak and positive	Araya et al. 2020
Plasma HIV-1 RNA load between 1.7log10 and 3log10 copies/ml	2.2	1.1, 4.3	Moderate and positive	Yitbarek et al. 2019
≥ 3log10 copies/ml	7.5	2.6, 21.5	Strong and positive	Yitbarek et al. 2019
Impairment in the activity of daily living	7.19	1.73, 21.83	Strong and positive	Belete et al. 2017
Have no communication about safe sexual intercourse	2.88	1.61, 5.16	Moderate and positive	Wubetu et al. 2021
Having co-morbid opportunistic infection	7.48	4.1, 13.64	Strong and positive	Mossie et al. 2014
Having comorbid depression and anxiety	5.51	1.81, 16.79	Strong and positive	Wubetu et al. 2021
Higher duration of HIV illness	1.01	1.001, 1.02	Weak and positive	Wubetu et al. 2021
Late clinical stage of the illness	4.2	1.19, 14.44	Strong and positive	Belete et al. 2017
Advancing stages of the disease	3.558	1.41, 9.01	Strong and positive	Animut et al. 2019
Clinical stage III of the disease	5.6	1.7, 19.2	Strong and positive	Yitbarek et al. 2019

The pooled odds ratio of older people among the above mentioned studies was 2.97 (95% CI 2.12, 3.82). This implied that people with old age were 2.97 times at higher risk of developing HAND than people with 18–25 years old. In addition, the pooled odds ratio for illiterate people for the three studies reported above was found to be 4.20 (95% CI 3.12, 5.28). This showed that illiterate participants were 4.20 times more likely to develop HAND than participants with primary and secondary education. Substance use was also an associated factor for the development of HAND with a pooled estimate odds ratio of 6.28 (95% CI 0.73, 11.82). In addition, the advanced stage of the disease was also found to have a significant association with the development of HAND with an estimated pooled odds ratio of 4.45 (95% CI 3.16, 5.74) (Table 5).

**Table 5:** Pooled odds ratio of HAND among people with HIV

Factors	Estimates		Heterogeneity		Studies pooled
	Pooled effect size	95% CI	I 2	P-value	
Older age	2.97	2.12, 3.82	89.9%	< 0.001	[15, 27–30]
Being illiterate	4.20	3.12, 5.28	94.7%	< 0.001	[11, 15, 27]
Substance abuse	6.28	0.73, 11.82	99.8%	< 0.001	[11, 15, 28, 30]
Advanced stages of the diseases	4.45	3.16, 5.74	95.2%	< 0.001	[12, 21, 30]

## Discussion

To our knowledge, this is the first study to systematically search, select, and analyze the prevalence of HAND in Ethiopia, which was examined across 8 studies including 3529 participants. The pooled estimated prevalence of HAND was 39.15%. Old age, lack of education, substance use and advanced stage of the disease were the main determinants of HAND.

The overall pooled prevalence of HAND in Ethiopia was 39.15 (95% CI 29.36, 48.94). This finding was higher than the prevalence estimated in a systematic review and meta-analysis study conducted in sub-Saharan Africa among those on ART for 6 months was 30.39% (95% CI 13.17–47.61%) [10]. Nevertheless, some studies have verified that this pooled prevalence was lower than that in studies conducted in different countries such as 42.37% (95% CI 32.18, 52.56%) in sub-Saharan Africa pre-ART HIV patients [10], and42.6% (95% CI 39.7–45.5) in a global meta-analysis study [32]. This difference in findings may reflect differences in participants’ characteristics, sample sizes and publication years. This review included People with HIV who had ART care follow-up in health institutions but other studies may include those people with HIV who did not have ART care follow-up. In addition, developing countries, including those in sub-Saharan Africa, are currently undergoing a demographic and epidemiological transition and the impact of population aging in sub-Saharan Africa will increase the burden of non-communicable and degenerative diseases in this region [33].The discrepancy may also be due to the screening tools. It is worth noting that the vast majority of the studies used the IHDS, which has shown by Milanini et al. [34] to be very poorly sensitive in a multi country East African population (Uganda, Kenya and Tanzania). The reliance of the IHDS in so many of these studies is a critical caveat for the overall meta-analysis; likely the meta-analysis estimate is too high.

In this systematic review and meta-analysis, random effect models have been used bearing in mind the chances of substantial heterogeneity between studies which were confirmed with the I2 test. In the subgroup analysis, the prevalence of HAND was slightly higher in studies published from 2019 to 2021 years; 43.74% than studies from 2014 to 2017 years; 31.50%.

The pooled prevalence revealed clear differences in the prevalence of HAND among regions; studies from SNNPR reported a high prevalence of HAND. This discrepancy might be because of the cultural differences among regions and diverse measurement tools might attribute to the difference in the prevalence of HAND among these regions.

The prevalence of HAND was 40.98% for studies that used a sample size of more than 400 than for studies that used a sample size of less than 400 (36.10%). This difference needs further investigation because the higher the sample size should yield the lower the prevalence rate. However, in this case, the higher sample size yields a higher prevalence, and the lower sample size yields a lower prevalence.

In this study, aged people were three times at higher risk of developing HAND than people with 18–25 years. This finding was inconsistent with a meta-analysis study conducted in Sub-Saharan Africa [10]. The strong association observed in our study might reflect an increased vulnerability to HAND which may be intensified by lifestyle changes and physical changes during old age.

Aged people were about four times more likely to have HAND as compared to people with HIV who had primary and secondary education. This finding was supported by another study [34]. This could be due to literacy is significantly associated with virtually all neuropsychological measures, even though the correlation between education and neuropsychological test scores depends on the specific test. The impact of literacy is reflected in different spheres of cognitive functioning. Learning to read reinforces and modifies certain fundamental abilities, such as verbal and visual memory, phonological awareness, and visuospatial and visuomotor skills. Functional imaging studies are now demonstrating that literacy and education influence the pathways used by the brain for problem-solving. The existence of partially specific neuronal networks as a probable consequence of the literacy level supports the hypothesis that education impacts not only the individual's day-to-day strategies but also the brain networks [34].

People with HIV who used substances were six times more likely to have HAND as compared to people with HIV who didn’t use the substance. The current study was consistent with another study [35]. HIV-associated neurocognitive impairment was associated with problematic methamphetamine use and higher plasma HIV RNA levels [35].

Moreover, the current study revealed that an advanced stage of the diseases or clinical stage III of HIV was 4.45 times more likely to have HAND than clinical stage I HIV. This might be because clinical stage III of HIV may affect the central nervous system and may expose the individual to develop different kinds of neurocognitive disorders.

### Limitations

However, several limitations exist in this study. Firstly, screening tools were used to measure HAND and the same cut-off values of each screening tool were not used in all studies. So, we should cautiously apply the results to the population. Secondly, all of the included studies were performed in Ethiopia, which significantly affects the Africa representativeness of the estimates. Thirdly, a small number of studies were used in our subgroup analysis which may reduce the precision of the estimate. Fourthly, the researchers did not use a standard definition of neurocognitive impairment.

## Conclusion

In this meta-analysis, the pooled prevalence of HAND in Ethiopia was high. Older age, substance abuse, advanced stages of the diseases, and having no education were the main determinants for HAND in Ethiopia. Therefore, based on our conclusions, health education and early screening of people living with HIV as well as training of health professionals working in the hospital on HAND are highly recommended.

## Data Availability

All data generated or analyzed during this study are included in this manuscript.

## Abbreviations

AIDS: Acquired Immune Deficiency Syndrome
AOR: Adjusted odds ratio
ART: Antiretroviral therapy
IDHS: International HIV dementia scale
HAND: HIV associated neurocognitive disorders
HIV: Human immunodeficiency virus
MMSE: Mini-Mental State Examination tool
PRISMA: Preferred Reporting Items for Systematic Reviews and Meta-Analyses
SNNPR: Southern Nations, Nationalities, and Peoples’ Region
